# Effect of amisulpride on the expression of serotonin receptors, neurotrophic factor BDNF and its receptors in mice with overexpression of the aggregation-prone [R406W] mutant tau protein

**DOI:** 10.18699/vjgb-24-45

**Published:** 2024-07

**Authors:** Е.М. Кондаурова, A.A. Komarova, T.V. Ilchibaeva, A.Ya. Rodnyy, E.A. Zalivina, V.S. Naumenko

**Affiliations:** Федеральный исследовательский центр Институт цитологии и генетики Сибирского отделения Российской академии наук, Новосибирск, Россия; Федеральный исследовательский центр Институт цитологии и генетики Сибирского отделения Российской академии наук, Новосибирск, Россия; Федеральный исследовательский центр Институт цитологии и генетики Сибирского отделения Российской академии наук, Новосибирск, Россия; Федеральный исследовательский центр Институт цитологии и генетики Сибирского отделения Российской академии наук, Новосибирск, Россия; Федеральный исследовательский центр Институт цитологии и генетики Сибирского отделения Российской академии наук, Новосибирск, Россия; Федеральный исследовательский центр Институт цитологии и генетики Сибирского отделения Российской академии наук, Новосибирск, Россия

**Keywords:** Alzheimer’s disease, tau protein, amisulpride, 5-HT7 receptor, Cdk5 kinase, Bdnf, Ngfr, Ntrk2, mice, болезнь Альцгеймера, тау-белок, амисульприд, 5-НТ7-рецептор, киназа Cdk5, Bdnf, Ngfr, Ntrk2, мыши

## Abstract

Serotonin 5-HT7 receptors (5-HT7R) are attracting increasing attention as important participants in the mechanisms of Alzheimer’s disease and as a possible target for the treatment of various tau pathologies. In this study, we investigated the effects of amisulpride (5-HT7R inverse agonist) in C57BL/6J mice with experimentally induced expression of the gene encoding the aggregation-prone human Tau[R406W] protein in the prefrontal cortex. In these animals we examined short-term memory and the expression of genes involved in the development of tauopathy (Htr7 and Cdk5), as well as biomarkers of neurodegenerative processes – the Bdnf gene and its receptors TrkB (the Ntrk2 gene) and p75NTR (the Ngfr gene). In a short-term memory test, there was no difference in the discrimination index between mice treated with AAV-Tau[R406W] and mice treated with AAV-EGFP. Amisulpride did not affect this parameter. Administration of AAV-Tau[R406W] resulted in increased expression of the Htr7, Htr1a, and Cdk5 genes in the prefrontal cortex compared to AAV-EGFP animals. At the same time, amisulpride at the dose of 10 mg/kg in animals from the AAV-Tau[R406W] group caused a decrease in the Htr7, Htr1a genes mRNA levels compared to animals from the AAV-Tau[R406W] group treated with saline. A decrease in the expression of the Bdnf and Ntrk2 genes in the prefrontal cortex was revealed after administration of AAV-Tau[R406W]. Moreover, amisulpride at various doses (3 and 10 mg/kg) caused the same decrease in the transcription of these genes in mice without tauopathy. It is also interesting that in mice of the AAV-EGFP group, administration of amisulpride at the dose of 10 mg/kg increased the Ngfr gene mRNA level. The data obtained allow us to propose the use of amisulpride in restoring normal tau protein function. However, it should be noted that prolonged administration may result in adverse effects such as an increase in Ngfr expression and a decrease in Bdnf and Ntrk2 expression, which is probably indicative of an increase in neurodegenerative processes.

## Abstract (ru)

Серотониновые рецепторы 5-HT7 (5-HT7R) привлекают все больше внимания в качестве одного из важных звеньев в механизмах развития болезни Альцгеймера и возможной мишени для лечения различных тау-патологий. В настоящей работе исследовано влияние амисульприда (обратный агонист 5-HT7R) в модели экспериментального повышения экспрессии гена, кодирующего склонный к агрегации белок человека Tau[R406W], в префронтальной коре мышей линии C57BL/6J на кратковременную память и экспрессию генов, участвующих в развитии таупатии (Htr7 и Cdk5), а также биомаркеров нейродегенеративных процессов – гена Bdnf и его рецепторов TrkB (ген Ntrk2) и p75NTR (ген Ngfr). В тесте на кратковременную память мыши не было обнаружено разницы по индексу дискриминации между мышами, которым вводили AAV-Tau[R406W], и мышами с AAV-EGFP. Амисульприд не повлиял на данный показатель. Введение AAV-Tau[R406W] привело к повышению экспрессии генов Htr7, Htr1a и Cdk5 в префронтальной коре по сравнению с животными группы AAV-EGFP. При этом амисульприд в дозе 10 мг/кг у животных группы AAV-Tau[R406W] вызвал снижение уровня мРНК генов Htr7 и Htr1a по сравнению с животными группы AAV-Tau[R406W], которым вводили физиологический раствор. Выявлено снижение экспрессии генов Bdnf и Ntrk2 в префронтальной коре после введения AAV-Tau[R406W]. При этом амисульприд в различных дозах (3 и 10 мг/кг) вызывал такое же снижение транскрипции этих генов у мышей без таупатии. Интересно также, что у мышей группы AAV-EGFP после введения амисульприда в дозе 10 мг/кг повышался уровень мРНК гена Ngfr. Полученные данные позволяют рассматривать амисульприд в качестве агента для восстановления нормальной функции тау-белка. Однако следует учитывать возможный негативный эффект амисульприда при длительном применении, отражающийся в увеличении экспрессии гена Ngfr и снижении экспрессии генов Bdnf и Ntrk2, что может указывать на усиление нейродегенеративных процессов

## Introduction

It is well known that tau protein plays an important role in the maintenance of axonal structure and growth, as well as regulates the formation of neuronal polarity, axonal transport and neuroplasticity (Arendt et al., 2012). However, hyperphosphorylated tau protein loses its normal ability to stabilize microtubules in cells and aggregates in pathomorphological structures – paired helical filaments and neurofibrillary tangles (Grundke-Iqbal et al., 1986). This leads to dysfunction of tau protein and causes various tauopathies, including Alzheimer’s disease (AD).

Currently, more than 50 different pathogenic mutations of the MAPT gene encoding tau protein have been detected. Most of these mutations are in exons and occur in regions encoding the C-terminal microtubule-binding domain (Strang et al., 2019). Mutations in coding sequences are mostly missense mutations, although there are also data on deletions (Rovelet- Lecrux et al., 2009). The most common manifestations of these mutations are impaired binding to microtubules and, as a consequence, their dysfunction, while the effect on the tau protein aggregation in vivo is observed only for some mutations (Xia et al., 2019).

Tau[R406W] is one of the MAPT gene mutations that promotes protein aggregation due to the reduced ability of the phosphorylated form to bind to microtubules (Perez et al., 2000). This mutation (located in exon 13 of the MAPT gene) results in the replacement of arginine with tryptophan at position 406 (p.R406W) and causes familial frontotemporal lobar degeneration with tau pathology (FTLD-tau). The frequency of the p.R406W mutation is 0.62 % among patients with FTLD-tau and 0.26 % among patients with AD (Gossye et al., 2023). The location of this mutation near the MTBR (microtubule-binding region) may affect the ability of this region to cause conformational changes in the neighboring MTBR (Xia et al., 2019). An important fact is that the R406W mutation is located near to key amino acid residues (Ser396, Ser404) that are phosphorylated in tau protein during the formation of pathological paired helical filaments (Hutton et al., 1998).

On the other hand, it is known that the brain serotonin (5-HT) system also plays an important role in the pathological development and clinical manifestations of primary tauopathies, including frontotemporal dementia, progressive supranuclear palsy and corticobasal degeneration (Huey et al., 2006; Murley, Rowe, 2018). The function of the 5-HT system is realized through numerous receptors. Nowadays, there is a growing number of studies investigating the role of 5-HT receptors in the mechanisms of tauopathies and AD development (Eremin et al., 2023).

In this regard, the 5-HT7 receptor (5-HT7R) has attracted particular attention. Recent studies have demonstrated that the constitutive activity of 5-HT7R induces hyperphosphorylation of tau protein and its subsequent aggregation through interaction with CDK5 kinase. Moreover, administration of the highly selective 5-HT7R inverse agonist SB-269970 prevents receptor-induced accumulation and hyperphosphorylation of tau protein (Labus et al., 2021).

Also, it has been shown that amisulpride (a drug with antipsychotic, antidepressant and procognitive effects), a strong inverse agonist of 5-HT7R, is able to affect the hyperphosphorylation of tau protein. The therapeutic potential of amisulpride in preventing/dispersing tau aggregation and tau-mediated pathology has been confirmed in vitro (in Tau- BiFC HEK293 cells and in human cortical neurons with the Tau[R406W] mutation) and in vivo (in mice overexpressing human mutant Tau [R406W] protein in the prefrontal cortex, and in transgenic mice expressing human mutant Tau[P301L] protein). In these animal models of tauopathy, treatment with amisulpride prevented tau protein hyperphosphorylation, aggregation, and neurotoxicity, and reversed memory impairment in both mouse strains (Jahreis et al., 2023).

In addition, it was shown that chronic administration of amisulpride in OXYS rats (a model of sporadic AD) (Stefanova et al., 2015) reduced phosphorylation of tau protein in the cortex and hippocampus of 3-month-old animals (Molobe-kova et al., 2023). Besides, in the hippocampus of 1- and 3-month-old rats, amisulpride also reduced the mRNA level of the Cdk5 kinase gene (Molobekova et al., 2023).

It is well known that the progression of tauopathies and AD causes the development of nerve cells atrophy in the cerebral cortex, hippocampus and other subcortical structures (Bettens et al., 2010). Thus, it was shown that the Tau[R406W] mutation causes disturbances in genes associated with neurogenesis and synaptic function in mouse neurons (Minaya et al., 2023). Among the biomarkers of neurodegenerative processes, the brain-derived neurotrophic factor (BDNF) is well known. The decrease in BDNF mRNA and protein levels in the cerebral cortex and hippocampus was shown in AD (Hock et al., 2000). BDNF-induced neuronal growth and development are mediated by its receptors, tyrosine kinase receptor B (TrkB) and common neurotrophin receptor p75 (p75NTR), which bind with BDNF and proBDNF, respectively. Accumulating evidence indicates the cross-talk between 5-HT and BDNF, suggesting that both systems may control each other’s functions by acting through shared intracellular signaling pathways. Balance in the functioning of the 5-HT and BDNF systems appears to be fundamental for the development of a normal phenotype (Popova, Naumenko, 2019).

Thus, the aim of the study was to investigate the effects of amisulpride in mice with experimentally induced expression of the Tau[R406W] gene (using an adeno-associated viral construct in vivo) in the prefrontal cortex on short-term memory and on the expression of genes that are involved in the development of tauopathy (Htr7 and Cdk5), as well as the gene of BDNF and its receptors (Ntrk2 (encodes TrkB) and Ngfr (encodes p75NTR)).

## Materials and methods

Animals. Experiments were carried out on 2-month-old C57BL/6J male mice. Work with animals was performed at the Center for Genetic Resources of Laboratory Animals, Institute of Cytology and Genetics of the Siberian Branch of the Russian Academy of Sciences, under standard conditions of a conventional vivarium (grant of the Russian Ministry of Science and Higher Education No. RFMEFI62119X0023). Animals were kept and tested in accordance with the Instructions for the Care and Use of Laboratory Animals (National Institute of Health’s Guide for the Care and Use of Laboratory Animals, NIH Publications, 2010).

Plasmids. Plasmids carrying the Tau[R406W] and EGFP (EGFP – enhanced green fluorescent protein) genes or only EGFP under control of the synapsin promoter were obtained from Professor E.G. Ponimaskin (MHH, Hannover, Germany).

Cell transfection. Packaging of pAAV_SynH1-2_ Tau[R406W]-EGFP and pAAV_SynH1-2_EGFP plasmids to adeno-associated viral (AAV) capsids was performed by their co-transfection with AAV-DJ and pHelper plasmids (Cell Biolabs, Inc., USA ) into HEK293FT cells that were incubated according to the protocol described previously (Kondaurova et al., 2021). Viral particles were collected after 48 h according to the protocol described previously (Grimm et al., 2003). The number of viral particles obtained was determined by quantitative real-time PCR analysis and diluted to a concentration of 109 viral particles/μl.

Stereotactic injection. Before the procedure, the animals were anesthetized with a mixture of 2,2,2-tribromoethanol and 2-methyl-2-butanol and placed in a stereotaxic frame (TSE Systems, Germany). Briefly, the scalp was opened, and two holes were drilled in the skull: AP: +1.5 mm, LR: ±1 mm, DV: 1 mm (http://labs.gaidi.ca/mouse-brain-atlas/?ml= 1.5&ap=-2&dv=2). Mice of both groups (36 males “AAVTau[ R406W]” and “AAV-EGFP”) were bilaterally injected with the AAV-Tau[R406W]-EGFP or AAV-EGFP viral construct into the prefrontal cortex. After the bilateral injections of the virus, the incision was closed with interrupted silk sutures, and the animal was placed in a warm cage and monitored closely (Kondaurova et al., 2021).

Pharmacological administration. Seven days after AAV administration, each group was divided into three subgroups (12 mice per subgroup). The effect of chronic amisulpride administration (Sanofi-Aventis, France) was assessed after 4 weeks of intraperitoneal administration in the doses of 3 and 10 mg/kg for the first and second subgroups, respectively, in a volume of 10 μl/g. Animals of the third subgroup were treated with the same volume of saline. Two days before the experiments, mice were placed in individual cages to remove the group effect

“Open field” test. This test was carried out in a circular arena (40 cm in diameter) surrounded by a white plastic wall (25 cm high) and illuminated through a mat and semitransparent floor with two halogen lamps of 12 W each placed 40 cm under the floor (Kulikov et al., 2008). Each mouse was placed near the wall and tested for 5 min. The animal’s behavior was recorded for 5 minutes using a camera located at a distance of 80 cm from the arena. The arena was treated with 70 % alcohol after each test. The video stream from the camera was analyzed frame by frame using the original EthoStudio software (Khotskin et al., 2019). The path length was measured automatically (horizontal activity).

Short-term memory test (“recency test”). This test was performed within the framework of the “open field” test paradigm. At the first stage of the “recency test”, the animals were familiarized with two identical objects (plastic cubes measuring 5 × 5 cm – “old object”), located in the center of the arena at a distance of 8–10 cm from each other and 10 cm from the walls of the arena. Animals were tested for 10 min. 90 min after the first test, two other objects (plastic cups with a diameter of 4 cm and a height of 5 cm – “new object”) were presented. These objects were located in the center of the arena at a distance of 8–10 cm from each other and 10 cm from the walls of the arena. Animals were tested for 10 min. 90 min after the second stage, one of the presented objects was replaced with the first object (plastic cubes, 5 × 5 cm). The animal was tested for 10 minutes. The time required to approach the new and old objects was assessed. Then discrimination index was calculated using the formula: (time for the “new object” – time for the “old object”) / total time for both objects.

Dissection of brain samples. In 48 h after behavioral testing, animals were removed from the experiment by decapitation. Immediately after euthanasia, the brain was removed and the necessary brain structures (prefrontal cortex, hippocampus) were excised on ice, frozen in liquid nitrogen. Until further procedures, the structures were stored in a lowtemperature refrigerator at –80 °C.

Fluorescence Microscopy. At least 6 weeks after AAV injection, one or two mice from each group were transcardially perfused for 2 min with 20 mL of phosphate-buffered saline (PBS) and 20 mL of a 4 % paraformaldehyde solution for 10 min under anesthesia. The brain was removed and postfixed with 4 % paraformaldehyde for 16 h and immersed in 30 % sucrose in PBS for 2 days. Sequential 12 μm slices were prepared on a cryostat (Thermo Scientific, Germany). Cell nuclei were stained with a bis-benzimide solution (Hoechst 33258 dye, 5 μg/mL in PBS; Sigma-Aldrich, Germany). The sections were then mounted in antiquenching Fluoromount G medium (Southern Biotechnology Associates, USA) followed by examination using an Olympus IX83P2ZF confocal microscope.

RNA isolation. The brain structures were homogenized in 300 μl TRIzol Reagent (Life Technologies, USA) according to the manufacturer’s protocol. The total RNA was dissolved in 24.5 μl of water treated with diethyl pyrocarbonate (DEPC). To eliminate possible genomic DNA contaminations, 0.5 μl of DNase (RNase-free DNase, Promega, USA, 1,000 p. u./ ml) was added. The samples were incubated for 15 minutes at 37 °C, and then for 10 minutes at 65 °C. The RNA concentrations were determined using a Nanodrop2000c spectrophotometer (Thermo Fisher Scientific), and diluted to 125 ng/μl. The RNA was stored at –80 °C.

Real-time RT-PCR. The gene expression was determined using a quantitative reverse transcription-polymerase chain reaction (RT-PCR) developed in our laboratory (Kulikov et al., 2005; Naumenko, Kulikov, 2006; Naumenko et al., 2008). Two types of standards were used: external and internal. An internal standard (housekeeping genes Polr2a (RNA polymerase II gene) and B2m (β2-microglobulin gene)) was used to monitor reverse transcription and as a basis for calculating the mRNA levels of the target genes. Mouse DNA of a known concentration served as an external standard, which made it possible to control the PCR and determine the number of mRNA copies of the studied genes in the samples. To determine the mRNA levels, we used the ratio of the cDNA level of the studied genes to the geometric mean level of cDNA of the rPol2a and B2m genes.

Primers for cDNA amplification were selected based on sequences published in the EMBL nucleotide database and synthesized at the Bioset company (Novosibirsk, Russia). PCR was carried out on a Real-time CFX96 Touch cycler (Bio-Rad, USA) in accordance with the following protocol: 3 min at 95 °C; 40 cycles with three stages: 10 sec at 95 °C, 30 sec at the primer annealing temperature, 20 sec at 72 °C (Supplementary Material 1)1.


Supplementary Materials are available in the online version of the paper: https://vavilov.elpub.ru/jour/manager/files/Suppl_Kond_Engl_28_4.pdf


Statistical Analysis. Statistical analysis was performed using GraphPadPrism 9.1.0. To search and exclude outliers form the analysis, the ROUT method (Q = 0.05) was used. The normal distribution of samples was tested using the Kolmogorov–Smirnov and Shapiro–Wilk tests. According to these criteria, all data have normal distribution. To identify differences between groups, a two-way ANOVA with posthoc Fisher’s multiple comparison was carried out. The results were presented as m ± SEM (m – mean; SEM – standard error of the mean). The statistical significance value was set at р < 0.05.

## Results

**Fig. 1.**
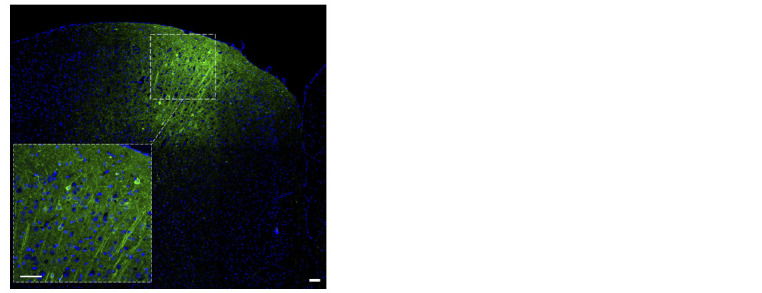
Microphotographs of the mouse prefrontal cortex section demonstrating successful AAV-mediated cell transfection as indicated by EGFP (enhanced green fluorescent protein) expression. Scale bar: 50 μm.

Fluorescence microscopy of mouse brain sections was used to verify the correct injection of the constructs. Brain sections of the prefrontal cortex area showed fluorescence (emission at 510 nm) when excited by light with a wavelength of 488 nm, which confirmed the successful expression of the viral construct into the brain structure (Fig. 1).

**Fig. 2.**
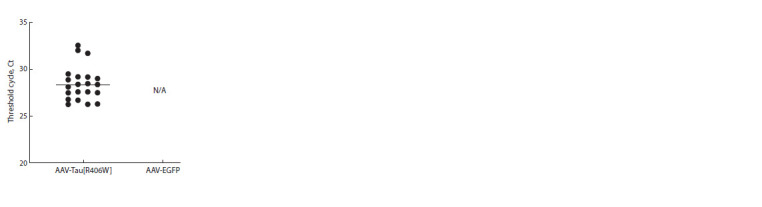
Expression (Ct) of the MAPT gene in the prefrontal cortex of mice overexpressing Tau[R406W].

Based on the presence of the MAPT gene product (on average at PCR cycle 28), we confirmed the gene expression in the prefrontal cortex of mice treated with the AAV-Tau[R406W] construct, while transcription of this gene was not observed in control mice (AAV-EGFP) (Fig. 2).

**Fig. 3.**
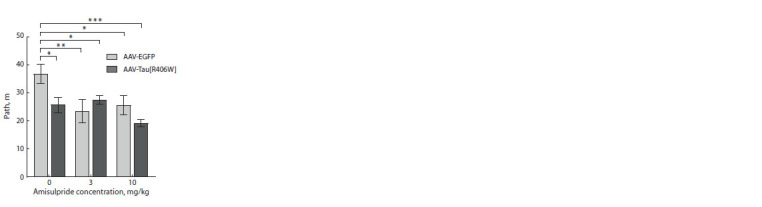
Changes in the motor activity of mice in the “open field” test * p <0.05; ** p < 0.01; *** p < 0.001.

In the “open field” test, the locomotor activity of mice was affected by both factors – the AVV construct (F1.57 = 3.598, p = 0.063) and the administration of amisulpride (F2.57 = = 4.580, p = 0.014), as well as by the interaction of these factors (F2.57 = 3.520, p = 0.036). AAV-EGFP animals showed increased locomotor activity not only compared to AAVTau[ R406W] mice ( p = 0.012), but also compared to AAVEGFP mice treated with amisulpride at the dose of 3 mg/kg ( p = 0.003) and 10 mg/kg ( p = 0.013) (Fig. 3).

**Fig. 4.**
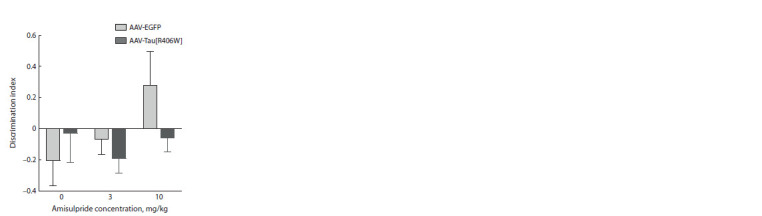
Effect of amisulpride on the discrimination index in the “recency test”.

Neither amisulpride (F2.26 = 1.8, p > 0.05), nor AAV administration (F1.26 = 1.111, p > 0.05) and their interaction (F2.26 = 1.6, p > 0.05) had a significant effect on the discrimination index values in the “recency test” (Fig. 4).

**Fig. 5.**
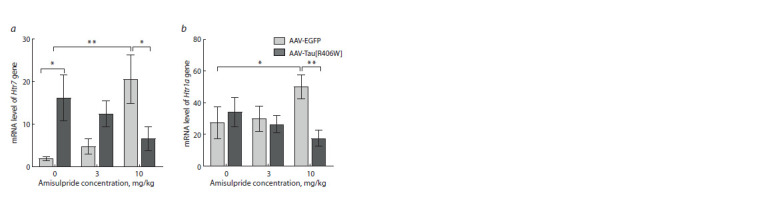
Effect of amisulpride on the transcription of the Htr7 (a) and Htr1a (b) genes in the prefrontal cortex of mice overexpressing Tau[R406W]. Values are normalized to the geometric mean of Polr2a and B2m mRNA. * p <0.05; ** p < 0.01.

A significant effect of interaction of AAV and amisulpride was found in the Htr7 mRNA level in the prefrontal cortex (F2.44 = 7.059, p = 0.002). Administration of the AAVTau[ R406W] construct caused an increase in the Htr7 gene transcription (p = 0.020). At the same time, we observed a restoration of the cortical Htr7 gene mRNA level to normal values in the mice from the AAV-Tau[R406W] group that were treated by amisulpride at the dose of 10 mg/kg (p = 0.014) (Fig. 5a). Amisulpride administration at the concentration of 3 mg/kg did not evoke a similar effect (p = 0.157). Interesting that when the drug was administered at the dose of 10 mg/ kg to AAV-EGFP mice the increased Htr7 mRNA levels was observed ( p = 0.004) (Fig. 5a).

For the Htr1a gene in the prefrontal cortex (Fig. 5b), similar differences were observed: the effect of interaction between the AAV and amisulpride factors (F2.52 = 3.359, p = 0.043) (Supplementary Material 2), a decrease in receptor gene transcription upon amisulpride treatment of AAV-Tau[R406W] mice (p = 0.005) at the dose of 10 mg/kg and an increase in the Htr1a mRNA levels in amisulpride (10 mg/kg) treated AAV-EGFP mice (p = 0.044).

**Fig. 6.**
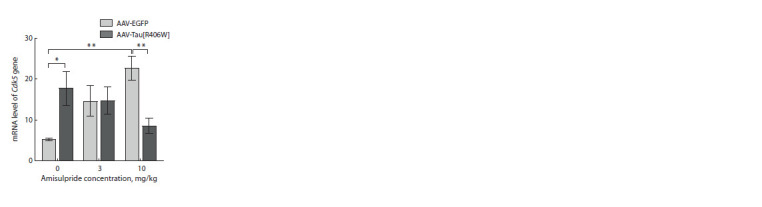
Effect of amisulpride on Cdk5 gene transcription in the prefrontal cortex of tau [R406W] overexpressing mice Values are normalized to the geometric mean of Polr2a and B2m mRNA. * p < 0.05; ** p <0.01.

When analyzing the Cdk5 gene mRNA level in the prefrontal cortex, an interaction of factors was found (F2.48 = 7.182, p = 0.002) (Supplementary Material 2). We showed that the Cdk5 mRNA level in mice from the AAV-Tau[R406W] group that were not exposed to amisulpride treatment was increased compared to the AAV-EGFP group (p = 0.021), while the effect of amisulpride at the dose of 10 mg/kg led to a decrease in Cdk5 gene expression (р = 0.004) compared to the AAVEGFP group. In addition, Cdk5 transcription was increased by 10 mg/ kg amisulpride in AAV-EGFP mice compared to AAV-EGFP saline-treated animals (p = 0.001) (Fig. 6).

We investigated the effect of amisulpride on the mRNA level of the brain-derived neurotrophic factor Bdnf and its receptors Ntrk2 (encodes the TrkB receptor) and Ngfr (encodes the p75NTR receptor). In the prefrontal cortex for Bdnf mRNA, the effect of AAV administration, amisulpride treatment and their interaction was observed. For the Ntrk2 gene, only AAV administration and the interaction of the AAV and amisulpride factors were found (Supplementary Material 2). Bdnf (p <0.001) and Ntrk2 (р < 0.001) mRNA levels were decreased by mutant Tau[R406W] overexpression compared with AAV-EGFP in saline-treated mice

**Fig. 7.**
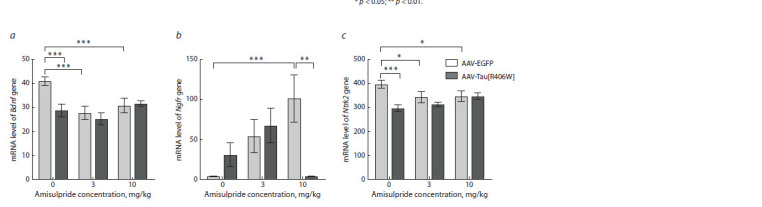
Effect of amisulpride on the transcription of the Bdnf (a), Ngfr (b) and Ntrk2 (c) genes in the prefrontal cortex of mice overexpressing Tau[R406W]. Values are normalized to the geometric mean of Polr2a and B2m mRNA. * p < 0.05; ** p < 0.01; *** p < 0.001.

In addition, a decrease in the level of Bdnf (р < 0.001) and Ntrk2 (р = 0.037) mRNAs was observed when the drug was administered at the dose of 3 mg/ kg to AAV-EGFP mice, as well as when amisulpride was administered at the dose of 10 mg/kg to AAV-EGFP mice (for Bdnf (p = 0.004) and Ntrk2 (p = 0.045)) (Fig. 7a, c). At the same time, the effect of interaction of the AAV and amisulpride treatment factors was observed for the Ngfr gene mRNA level in the prefrontal cortex (F2.43 = 4.752, p = 0.014) (Supplementary Material 2). The Ngfr gene expression in the cortex of AAV-EGFP mice increased upon administration of amisulpride at the concentration of 10 mg/kg (р = 0.002); however, mice overexpressing Tau[R406W] showed a decrease in the mRNA level of this gene when exposed to the same dose of the drug (р = 0.002) (Fig. 7b).

No statistically significant differences in the expression of all investigated genes were found in the hippocampus. In Supplementary Material 2, data from a two-factor analysis of variance are presented.

## Discussion

Here it was shown that locomotor activity in the “open field” test was reduced both in the AAV-Tau[R406W] group that received saline, and in groups that were treated by amisulpride at different doses compared to the AAV-EGFP group that received saline. This data are consistent with the results of the work by K. Jahreis et al.: they showed that administration of Tau[R406W] vector and treatment with amisulpride (1 mg/kg, 16 days) reduced locomotor activity in the “open field” test compared to control (Jahreis et al., 2023).

In the current study, amisulpride failed to produce a significant effect on the short-term memory of animals treated with AAV-Tau[R406W], in contrast to the paper of K. Jahreis et al., who showed an increase in the discrimination index in mice treated with AAV-Tau[R406W] and amisulpride (Jahreis et al., 2023). The lack of a significant amisulpride effect on short-term memory in our experiment may be due to a shorter recovery period after vector administration and before the beginning of amisulpride therapy. In our study, it was seven days, unlike the work of K. Jahreis et al., in which the recovery period took three weeks. Thus, the stage of tauopathy development is probably important for the amisulpride therapy of cognitive abilities

We found that administration of AAV-Tau[R406W] leads to increased mRNA levels of the Htr7 and Cdk5 genes in the prefrontal cortex compared to control animals. These findings are likely due to a neuroprotective response involving increased levels of 5-HT7R, which is known to be involved in the regulation of neuronal morphology, neurite outgrowth, dendritic spines, and synaptogenesis (Kobe et al., 2012). However, a recent study has shown a reduced Htr7 gene mRNA level in the anterior prefrontal cortex in postmortem brain samples from AD patients (Solas et al., 2021). This discrepancy can be explained by long-term neurodegenerative processes in the brains of AD patients, while in our work the effect of the mutant tau protein lasted only six weeks.

The increased transcription of the Cdk5 gene in AAVTau[ R406W] mice is consistent with a study of J. Labus and coauthors, who showed that CDK5 is responsible for the pathological effect of 5-HT7R on tau protein hyperphosphorylation (Labus et al., 2021). At the same time, the combined decrease in the mRNA levels of both Htr7 and Cdk5 in AAV- Tau[R406W] mice treated with amisulpride to values similar to those in control animals confirms the proposed mechanism of 5-HT7R inverse agonists action in restoring normal tau protein function in vivo. The increase of the Htr7, Htr1a and Cdk5 mRNA levels after amisulpride administration at the dose of 10 mg/kg in AAV-EGFP mice is probably a compensatory response to inhibition of the 5-HT7 receptor by amisulpride. The effect of amisulpride on the Cdk5 mRNA level is in good agreement with the data obtained on OXYS rats: in healthy one-month-old rats, amisulpride also increased the Cdk5 mRNA level in the cortex (Molobekova et al., 2023).

It is known that 5-HT1A (5-HT1AR) and 5-HT7R receptors can form heterodimers in vitro and in vivo. Such heterodimerization leads to agonist-mediated internalization of 5-HT1A receptors (Renner et al., 2012). Chronic activation of 5-HT7R causes desensitization of these receptors and also reduces the level and functional activity of 5-HT1A receptors in the frontal cortex, without affecting the level of 5-HT7R (Kondaurova et al., 2017). It has also been shown that overexpression of 5-HT7R in the midbrain leads to changes in 5-HT1AR gene expression depending on the mouse strain. In mice of the C57Bl/6J strain, a decrease in the 5-HT1AR gene mRNA level was detected in the frontal cortex, while in ASC (antidepressant sensitive cataleptics) mice, the expression of this gene was reduced in the hippocampus (Rodnyy et al., 2022). Amisulpride, as an inverse agonist of 5-HT7 receptors, suppresses receptor constitutive activity and, perhaps, can thus influence the mRNA levels of the 5-HT7R gene in a negative feedback manner. It is interesting to note that chronic administration of amisulpride at the dose of 10 mg/kg led to an increase in the expression of both the 5-HT7R gene and the 5-HT1AR gene, which may be due to the mutual regulation of these receptors through their heterodimerization.

BDNF is one of the most studied neurotrophic factors. It plays an important role in the growth and maturation of brain cells at all stages of development, and is involved in the regulation of synaptic transmission and plasticity in adulthood (Edelmann et al., 2015). In the context of AD, BDNF depletion is associated with tau protein phosphorylation and aggregation, Aβ accumulation, neuroinflammation, and neuronal death (Pisani et al., 2023). BDNF stimulation leads to dephosphorylation of tau protein through TrkB activation and phosphatidylinositol 3-kinase (PI3K) signaling (Elliott et al., 2005).

In our study, we found a decrease in the mRNA levels of BDNF and its receptor TrkB in the cortex after administration of AAV-Tau[R406W]. These data are in agreement with the decrease in the BDNF level observed in AD (Song et al., 2015). In addition, we showed that amisulpride administration at different doses also reduces the mRNA levels of these genes in both AAV-EGFP and AAV-Tau[R406W] mice. These results contradict previous findings indicating that amisulpride increases BDNF levels in human neuroblastoma SH-SY5Y cells (Park et al., 2011). However, there is evidence that amisulpride does not affect the Bdnf mRNA level in another cell model – in T98G glioma cells (Jóźwiak-Bębenista et al., 2017). The work of E.N. Rizos et al. also did not reveal any effect of amisulpride on the BDNF level in the blood serum of patients with schizophrenia (Rizos et al., 2010). At the same time, an increase in the expression and phosphorylation of TrkB was detected 30 min after activation of 5-HT7R (Samarajeewa et al., 2014).

On the one hand, it can be assumed that the mechanisms of amisulpride action in vitro and in vivo are different. On the other hand, it has been shown that in human neuroblastoma SH-SY5Y cells, the elongation of nerve fibers caused by incubation with 5-HT, nerve growth factor (NGF) or brainderived neurotrophic factor BDNF is blocked by 5-HT7R antagonists. The knockdown of the Htr7 gene also reduces the length of nerve fibers, whereas 5-HT7R activation by agonists increases the expression of the NGF and BDNF genes (Chang et al., 2022).

A recent paper by L.L. Shen and colleagues has shown that knockout of p75NTR receptor leads to a reduction in Aβ-induced tau hyperphosphorylation and neurodegeneration both in healthy mice and in a mouse model of human tauopathy, involving CDK5 and GSK3β kinases (Shen et al., 2019). These data suggest that p75NTR receptor at least partially mediates Aβ peptide-triggered tau pathology. However, in our study, overexpression of Tau[R406W] did not have a significant effect on the p75NTR receptor mRNA level. At the same time, we found that amisulpride increases transcription of the p75NTR receptor gene in AAV-EGFP mice. There are other literature data on the negative effects of long-term amisulpride administration through a decrease in choline ace-tyltransferase (ChAT). G.B. Huang et al. demonstrated that long-term amisulpride administration (45 days) in rats reduced the number of ChAT-positive cells in the prefrontal cortex but not in hippocampus, which may have a negative effect on cognitive function (Huang et al., 2012).

## Conclusion

Thus, the utilization of amisulpride in mice with Tau[R406W] overexpression led to a decrease in the Htr7 and Cdk5 genes mRNA level in the prefrontal cortex, which allowed us to suggest the drug as an agent for restoring normal tau protein function. However, the drug administration in mice without tauopathy caused a decrease in the Bdnf and Ntrk2 genes mRNA levels in the frontal cortex. At the same time, the levels of Htr7, Htr1a and Cdk5 mRNAs were increased in AAV-EGFP mice that were treated with the amisulpride. These changes probably reflect the negative effect of chronic amisulpride administration, which is also indirectly confirmed by an increase in the expression of the p75NTR receptor gene, which is known to initiate apoptotic processes in the brain.

## Conflict of interest

The authors declare no conflict of interest.
